# Supplementary material for: MolPLA: a molecular pretraining framework for learning cores, R-groups and their linker joints
Source: Bioinformatics. 2024 Jun 28;40(Suppl 1):i369–80. doi: 10.1093/bioinformatics/btae256 (PMC11211832; doi:10.1093/bioinformatics/btae256)
Supplement: btae256_Supplementary_Data [file btae256_supplementary_data.pdf]

Supplementary Material for **MolPLA:** *A Molecular Pre-training Framework for Learning Cores, R-Groups and their Linker Joints*

S1: Detailed statistics of the MolPLA pre-training dataset

Original GEOM: 304,466 molecules

1. After core extraction

(GEOM mol, core): 1,231,464 pairs

Molecule Core counts

: Distribution and percentile of core counts extracted from GEOM molecules

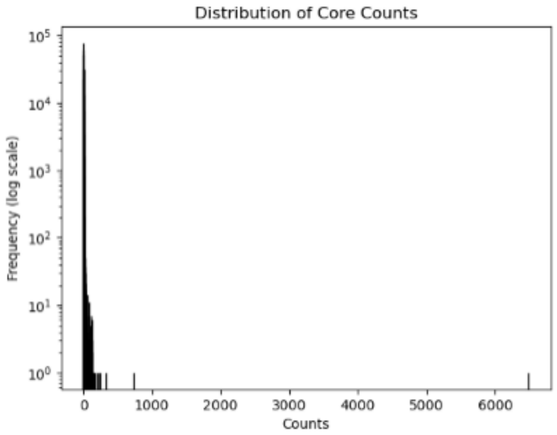

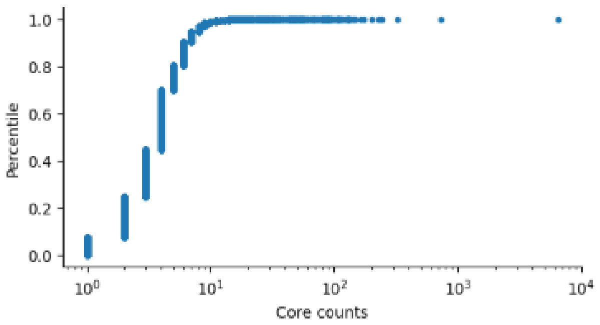

- Max: 6486, Min: 1, Median: 4, Mean: 4.05, Mode: 4

2. After applying core number threshold

(GEOM mol, core): 1,196,157 pairs

Apply a core number threshold of 10 for each molecule, randomly selecting cores for those with more than the threshold.

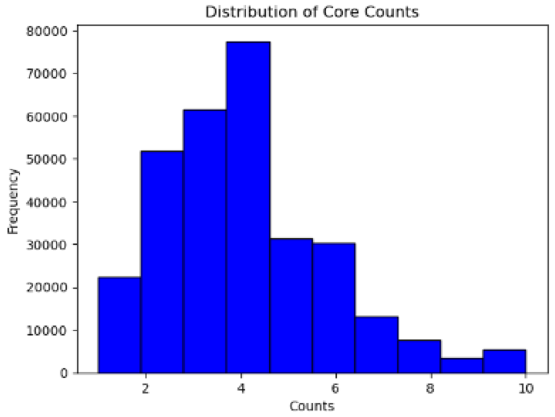

- Max: 10, Min: 1, Median: 4, Mean: 3.85, Mode: 4

3. After R-Group attachment

(GEOM mol, core, attached R-Groups): 1,259,946 pairs

Top 80 Putative Cores histogram

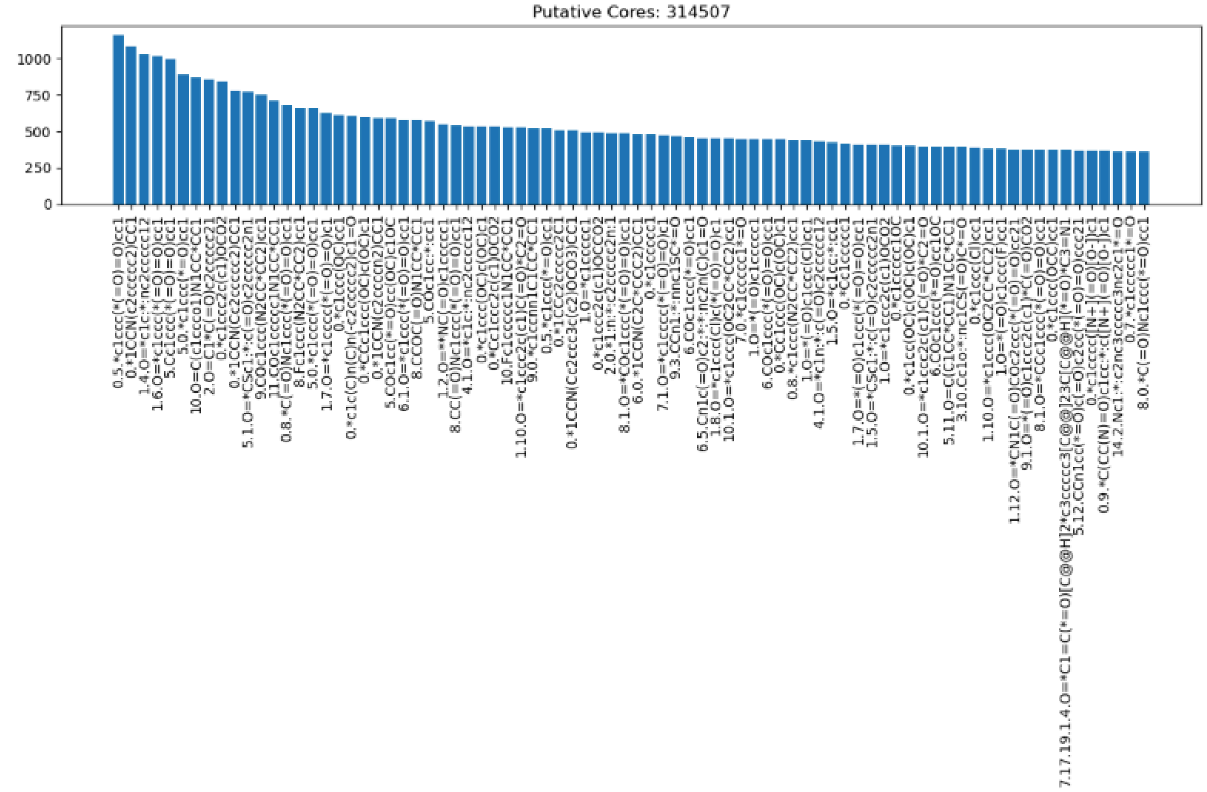

Top 20 Putative Cores image

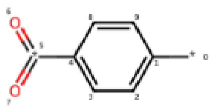

0.5.*c1ccc(*(=O)=O)cc1: 1164

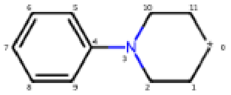

0.*1CCN(c2ccccc2)CC1: 1088

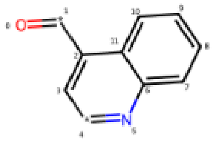

1.4.O=*c1c:*:nc2ccccc12: 1032

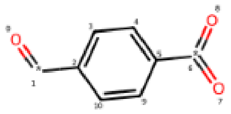

1.6.O=*c1ccc(*(=O)=O)cc1: 1017

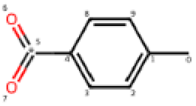

5.Cc1ccc(*(=O)=O)cc1: 994

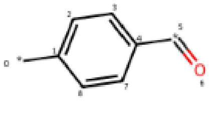

5.0.*c1ccc(*=O)cc1: 888

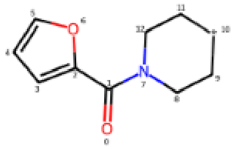

10.O=C(c1ccco1)N1CC*CC1: 868

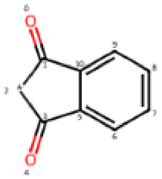

2.O=C1*C(=O)c2ccccc21: 857

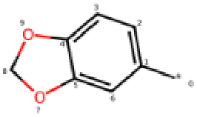

0.*c1ccc2c(c1)OCO2: 840

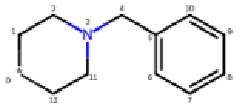

0.*1CCN(Cc2ccccc2)CC1: 779

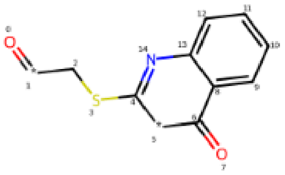

5.1.O=*CSc1:*:c(=O)c2ccccc2n1: 771

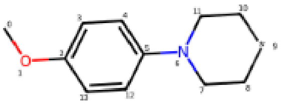

9.COc1ccc(N2CC*CC2)cc1: 753

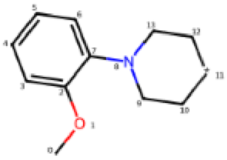

11.COc1ccccc1N1CC*CC1: 710

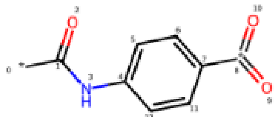

0.8.*C(=O)Nc1ccc(*(=O)=O)cc1: 681

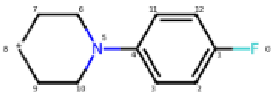

8.Fc1ccc(N2CC*CC2)cc1: 661

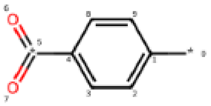

5.0.*c1ccc(*(=O)=O)cc1: 657

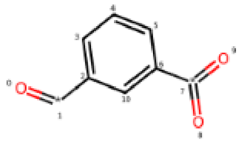

1.7.O=*c1cccc(*(=O)=O)c1: 624

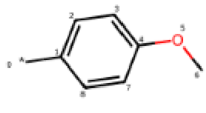

0.*c1ccc(OC)cc1: 610

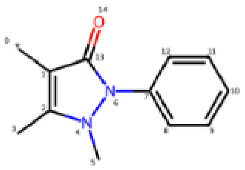

0.*c1c(C)n(C)n(-c2ccccc2)c1=O: 605

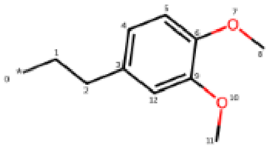

0.*CCc1ccc(OC)c(OC)c1: 598

Top 80 R-Groups histogram

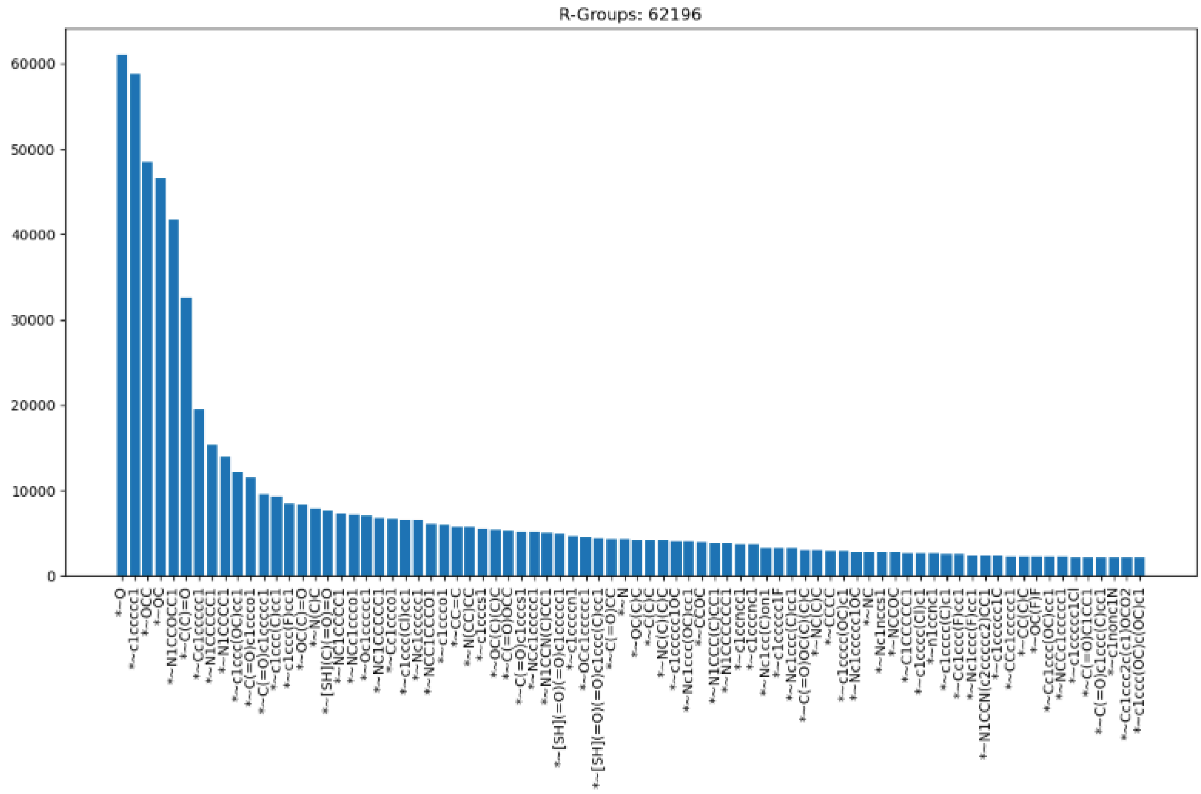

Top 20 R-groups image

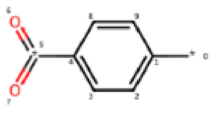

0.5.*c1ccc(*(=O)=O)cc1: 1164

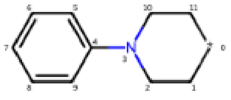

0.*1CCN(c2ccccc2)CC1: 1088

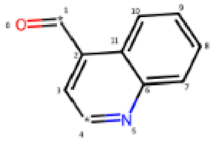

1.4.O=*c1c:*:nc2ccccc12: 1032

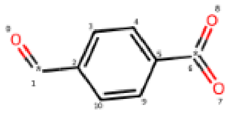

1.6.O=*c1ccc(*(=O)=O)cc1: 1017

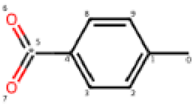

5.Cc1ccc(*(=O)=O)cc1: 994

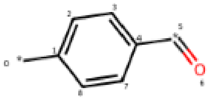

5.0.*c1ccc(*=O)cc1: 888

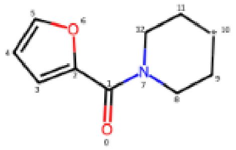

10.O=C(c1ccco1)N1CC*CC1: 868

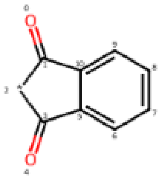

2.O=C1*C(=O)c2ccccc21: 857

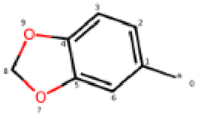

0.*c1ccc2c(c1)OCO2: 840

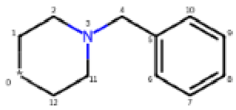

0.*1CCN(Cc2ccccc2)CC1: 779

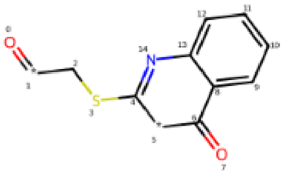

5.1.O=*CSc1:*:c(=O)c2ccccc2n1: 771

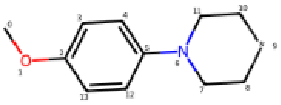

9.COc1ccc(N2CC*CC2)cc1: 753

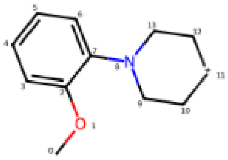

11.COc1ccccc1N1CC*CC1: 710

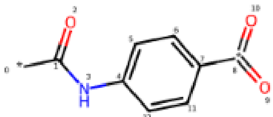

0.8.*C(=O)Nc1ccc(*(=O)=O)cc1: 681

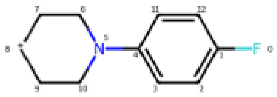

8.Fc1ccc(N2CC*CC2)cc1: 661

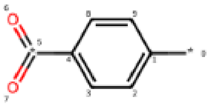

5.0.*c1ccc(*(=O)=O)cc1: 657

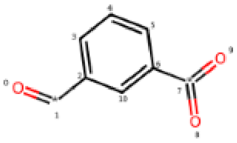

1.7.O=*c1cccc(*(=O)=O)c1: 624

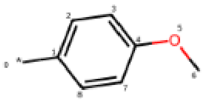

0.*c1ccc(OC)cc1: 610

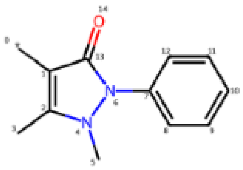

0.*c1c(C)n(C)n(-c2ccccc2)c1=O: 605

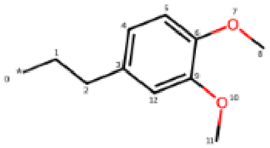

0.*CCc1ccc(OC)c(OC)c1: 598

Linker Atom Counts

: linker atom count histogram

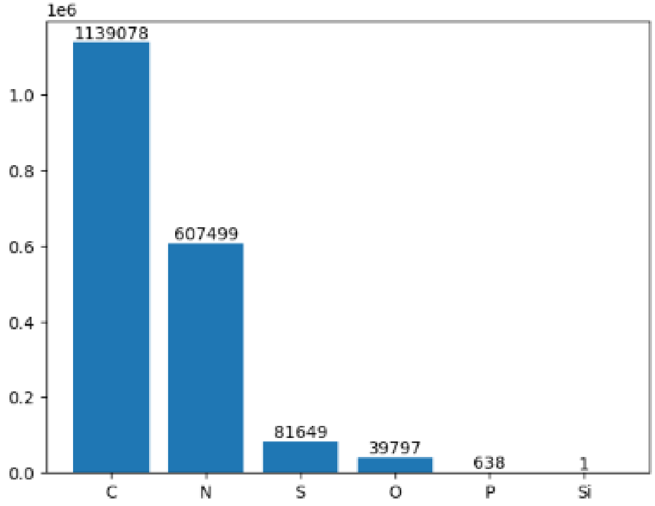

Linker Counts

: histogram of linker counts in a molecule

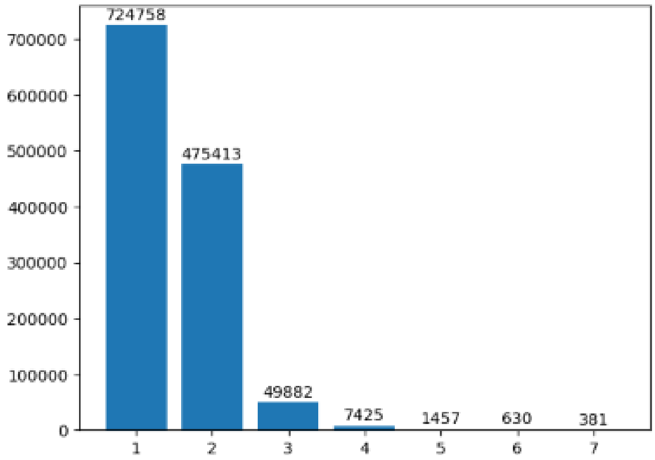

4. After R-Group attachment + common R-Groups filtering (reduce long tail)

(GEOM mol, core, attached R-Groups): 1,054,787 pairs

Filtering

- 99.99 percentile R-Groups = common R-Groups
- if more than half of total attached R-Groups common → filter out

Top 80 Putative Cores histogram

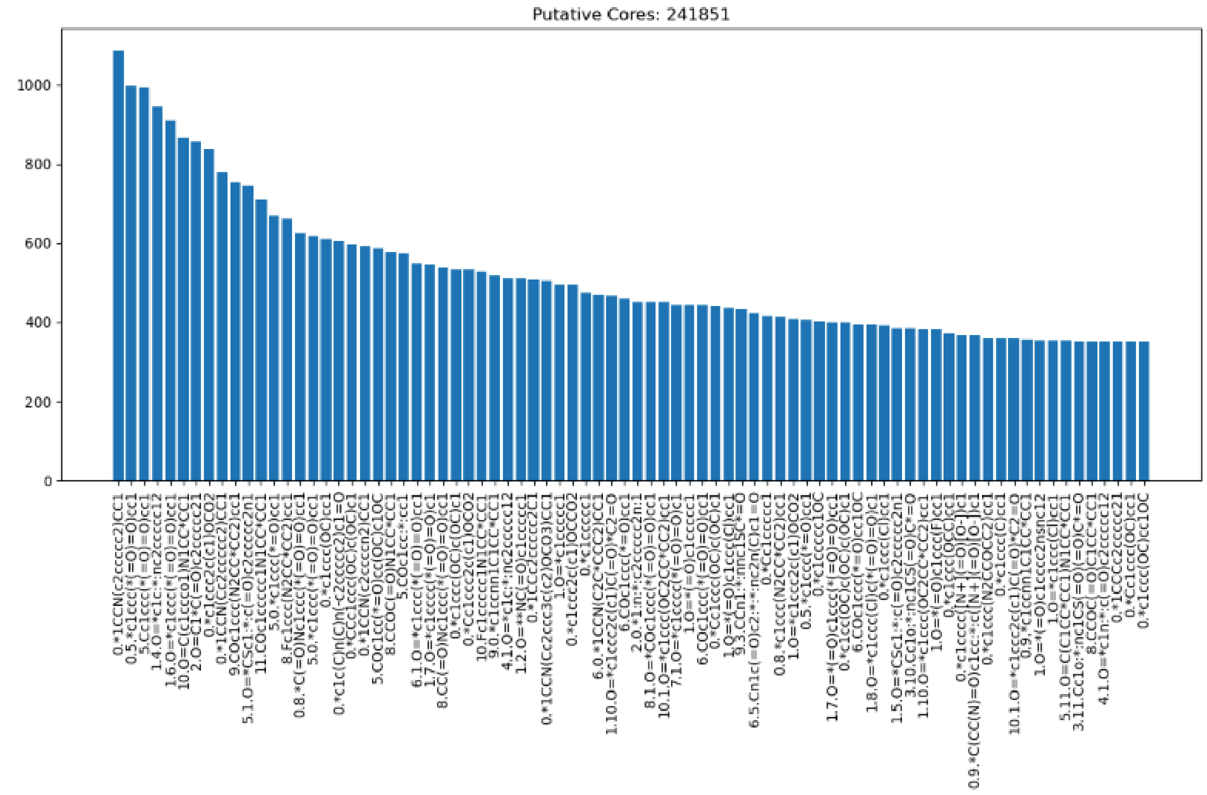

Top 20 Putative Cores image

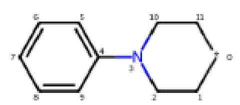

0.*1CCN(c2ccccc2)CC1: 1088

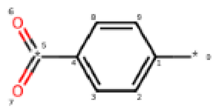

0.5.*c1ccc(*(=O)=O)cc1: 998

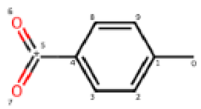

5.Cc1ccc(*(=O)=O)cc1: 994

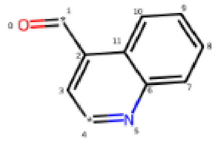

1.4.O=*c1c:*:nc2ccccc12: 946

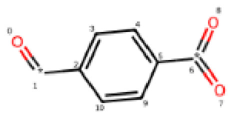

1.6.O=*c1ccc(*(=O)=O)cc1: 911

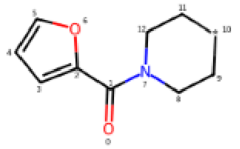

10.O=C(c1ccco1)N1CC*CC1: 867

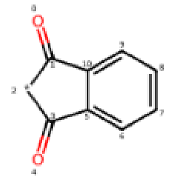

2.O=C1*C(=O)c2ccccc21: 857

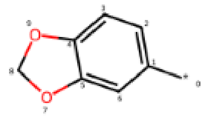

0.*c1ccc2c(c1)OCO2: 838

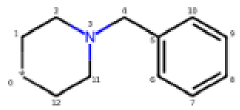

0.*1CCN(Cc2ccccc2)CC1: 779

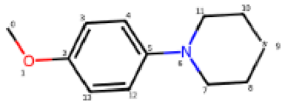

9.COc1ccc(N2CC*CC2)cc1: 753

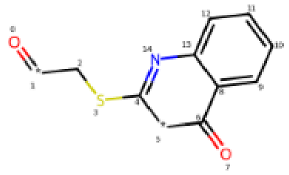

5.1.O=*CSc1:*:c(=O)c2ccccc2n1: 746

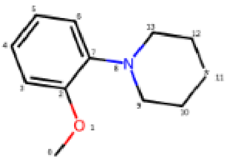

11.COc1ccccc1N1CC*CC1: 709

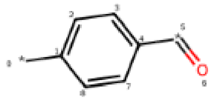

5.0.*c1ccc(*=O)cc1: 669

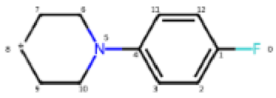

8.Fc1ccc(N2CC*CC2)cc1: 661

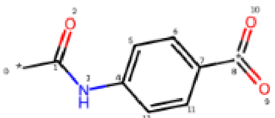

0.8.*C(=O)Nc1ccc(*(=O)=O)cc1: 626

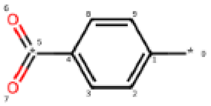

5.0.*c1ccc(*(=O)=O)cc1: 618

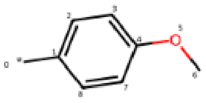

0.*c1ccc(OC)cc1: 609

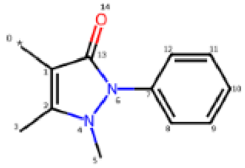

0.*c1c(C)n(C)n(-c2ccccc2)c1=O: 605

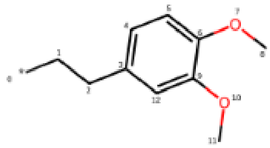

0.*CCc1ccc(OC)c(OC)c1: 598

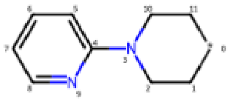

0.*1CCN(c2ccccn2)CC1: 592

Top 80 R-Groups histogram

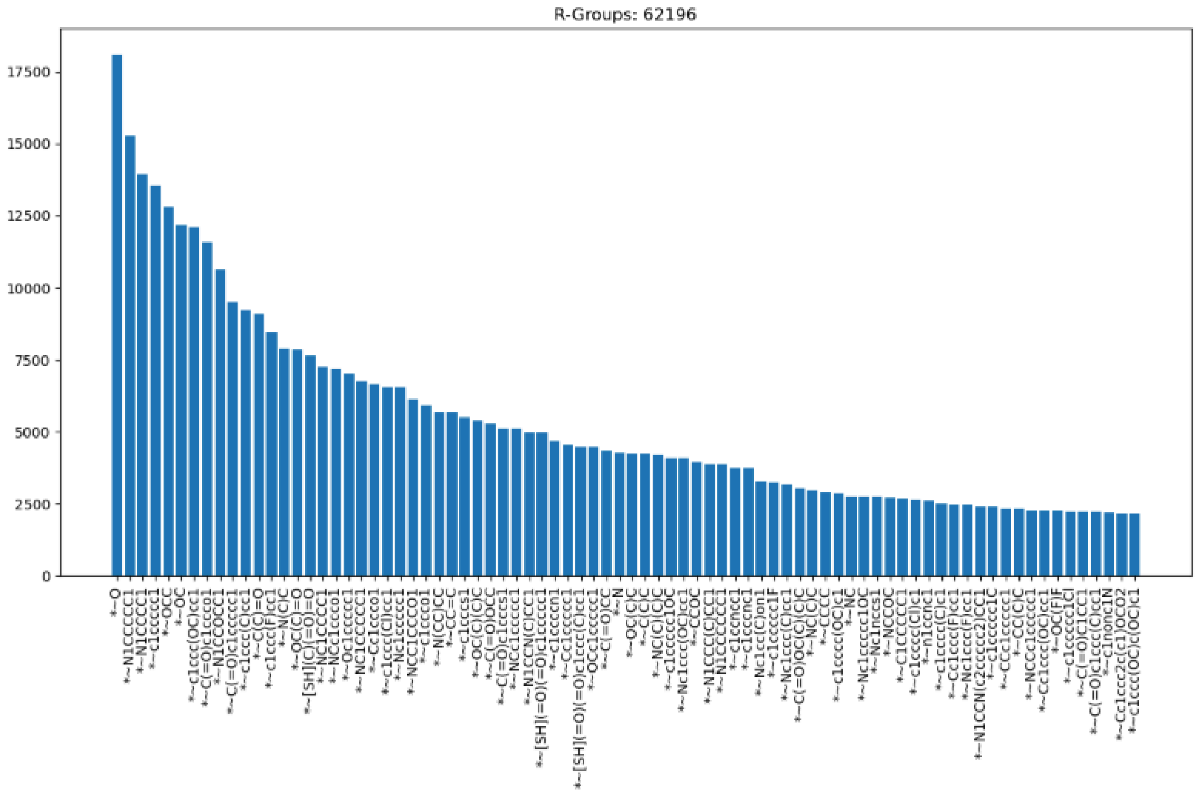

Top 20 R-Groups image

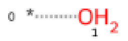

*~O: 18088

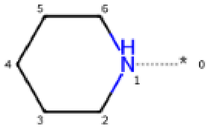

*~N1CCCCC1: 15272

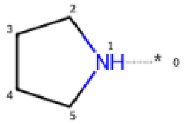

*~N1CCCC1: 13937

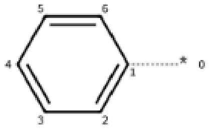

*~c1ccccc1: 13563

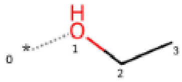

*~OCC: 12802

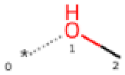

*~OC: 12189

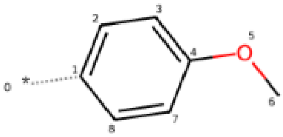

*~c1ccc(OC)cc1: 12105

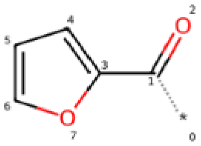

*~C(=O)c1ccco1: 11587

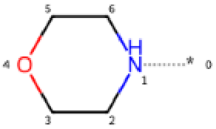

*~N1CCOCC1: 10643

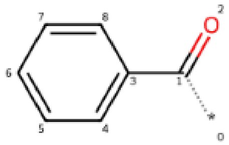

*~C(=O)c1ccccc1: 9504

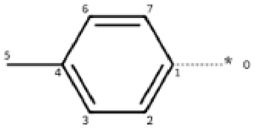

*~c1ccc(C)cc1: 9239

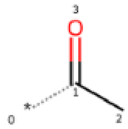

*~C(C)=O: 9098

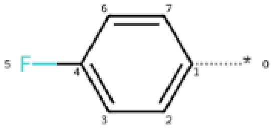

*~c1ccc(F)cc1: 8440

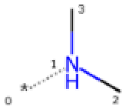

*~N(C)C: 7888

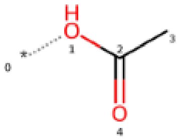

*~OC(C)=O: 7859

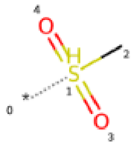

*~[SH](C)(=O)=O: 7653

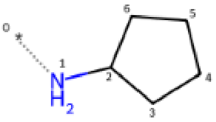

*~NC1CCCC1: 7244

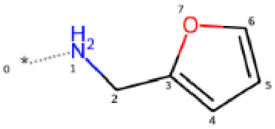

*~NCc1ccco1: 7203

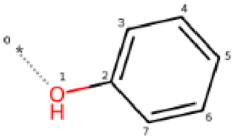

*~Oc1ccccc1: 7028

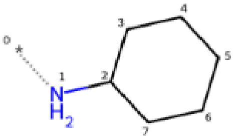

*~NC1CCCCC1: 6764

Compare Putative Cores

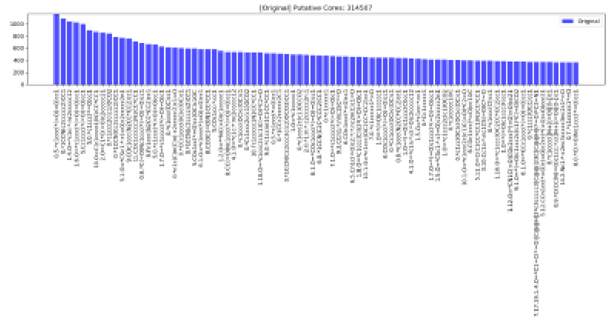

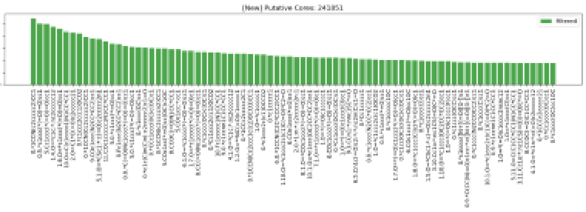

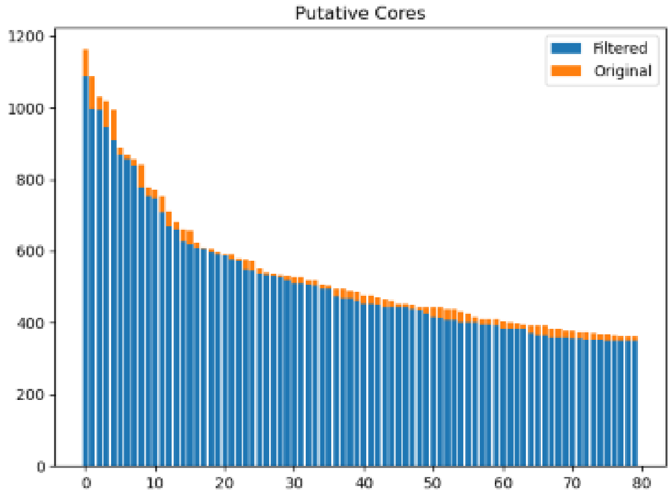

Compare R-Groups

⇒ reduced long tail

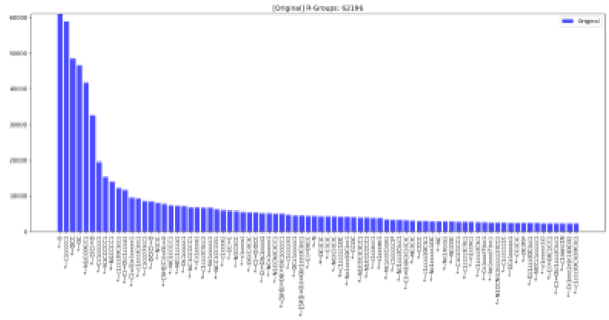

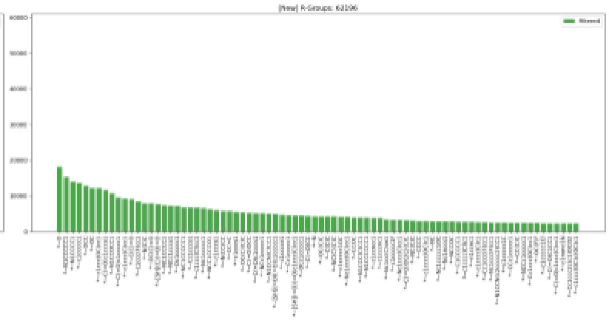

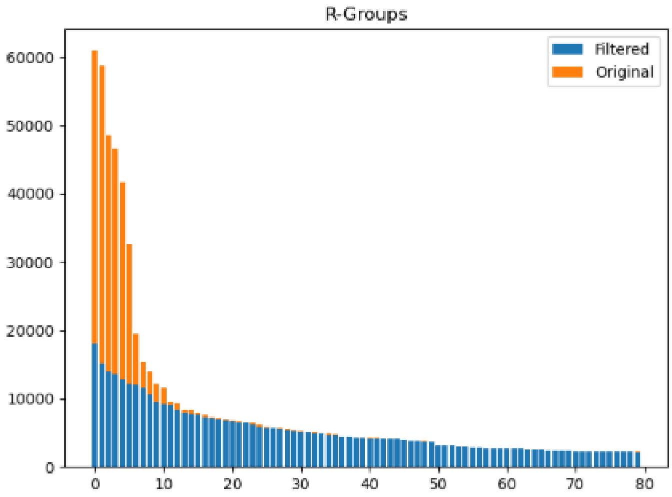

S2: Comparison between two molecular graph decomposition methods using Mucko Scaffolds and Naveja's Putative Core framework

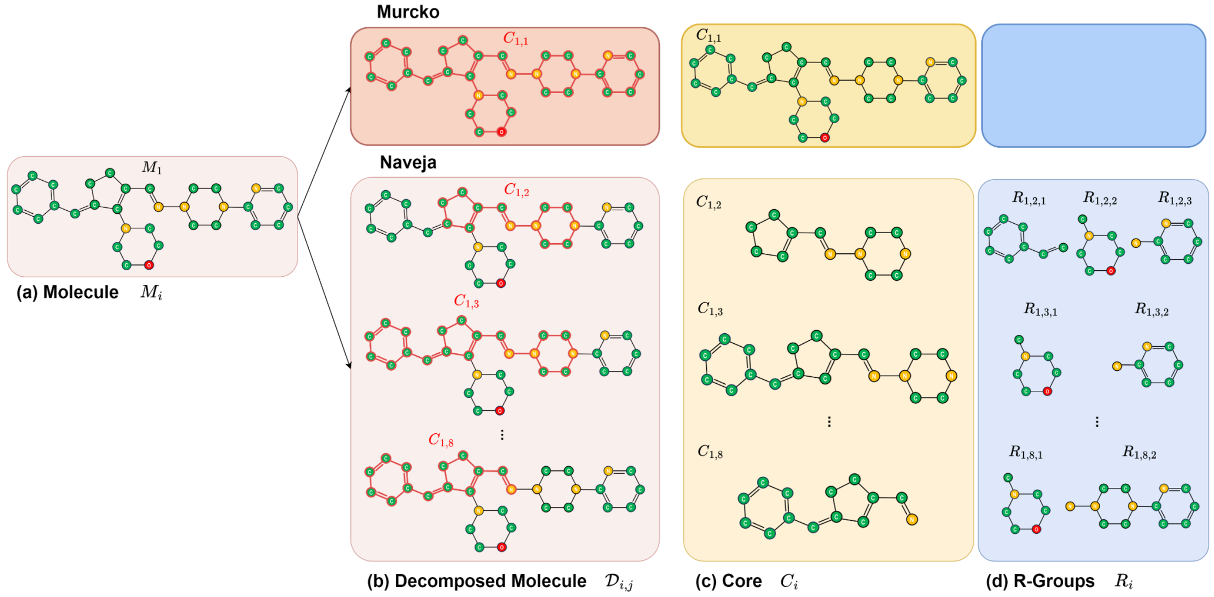

S3: Preprocessing Pipeline for the MolPLA pre-training dataset

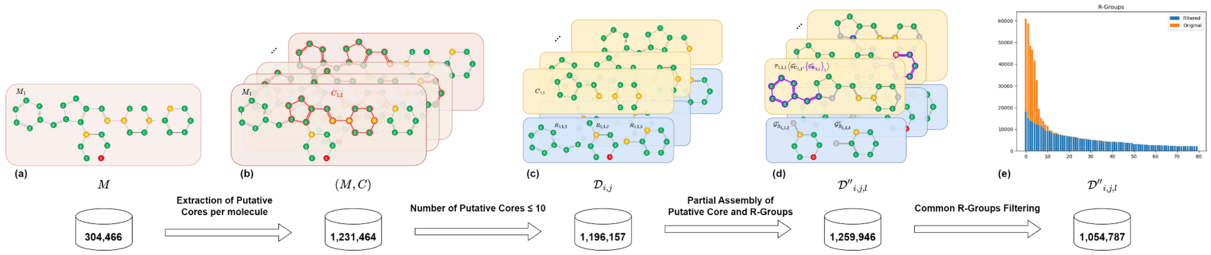

S4: Detailed illustration for our proposed molecular graph decomposition

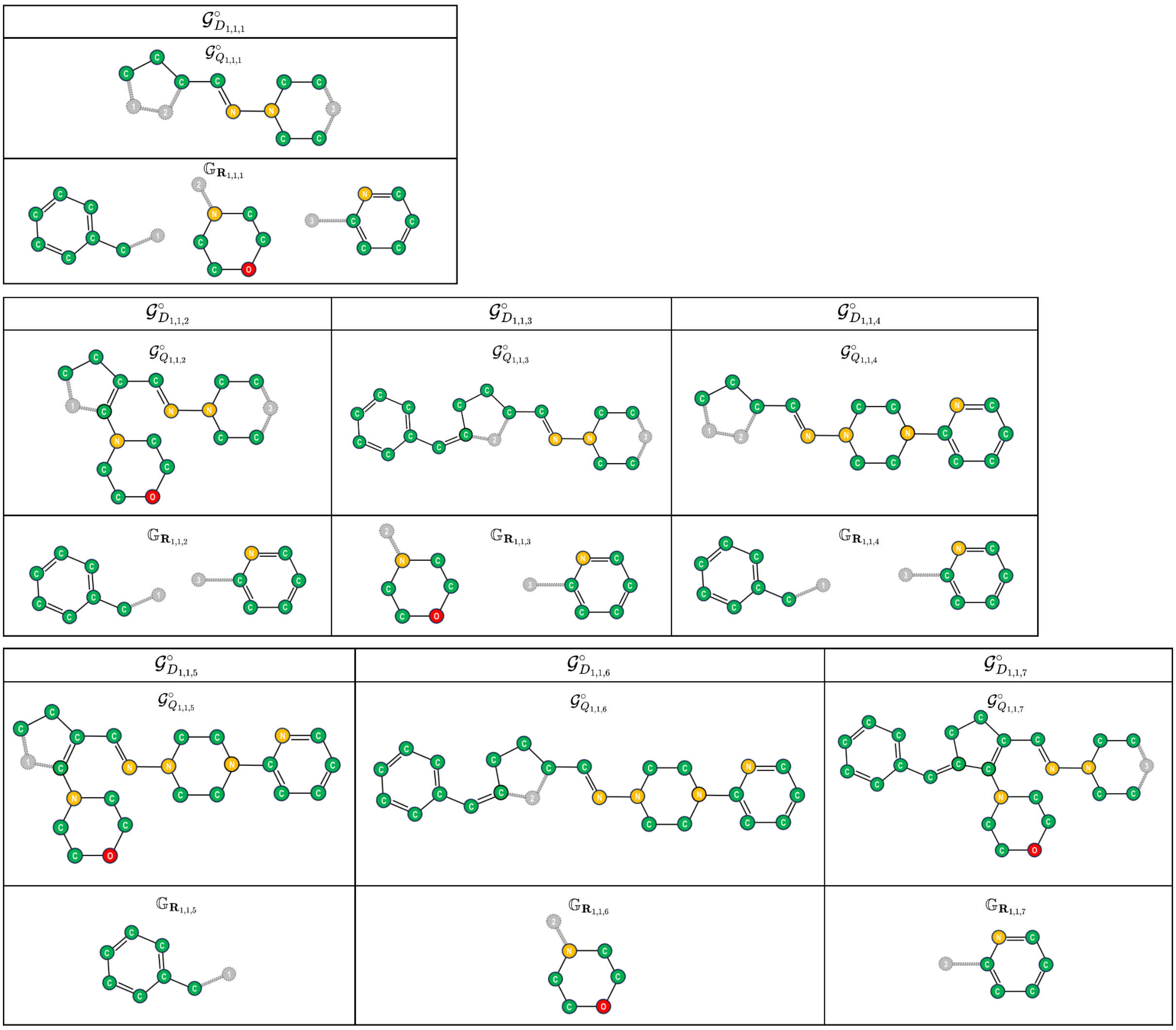

S5: Implementation details for MolPLA

The weight-sharing GNN-based **graph encoder** f_θ used in both MolPLA's Masked Graph Contrastive Learning and R-Group Retrieval Framework consists of five GIN layers followed by Layer Normalization applied to the updated node representations. The initial node and edge representations from graph input $\mathcal{G}$ are a summation of attribute-wise embeddings based on predefined molecular information obtained from Rdkit which are the following,

Attribute Type	Attribute Name	Number of Features
Node (Atom Features)	Atomic Number	128
	Formal Charge	11
	Chirality Tag	9
	Hybridization	9
	Number of Explicit Hs	9
	Aromaticity	2
Edge (Bond Features)	Conjugation	2
	Bond Type	22
	Bond Direction	7
	Bond Stereochemistry	6
	Aromaticity	2

The GIN layer updates node representations based on its neighbor information. The update rule for node v in the lth layer is mathematically expressed as follows,

$$h_v^{(l+1)} = \mathrm{MLP}^{(l)}\left((1+\epsilon^{(l)}) \cdot h_v^{(l)} + \sum_{u \in \mathcal{N}(v)} h_u^{(l)}\right)$$

where $h_v^{(l)}$ is the feature vector of node v at layer l, $\mathcal{N}(v)$ denotes the set of neighbors of v, $\mathrm{MLP}^{(l)}$ is a multi-layer perceptron at layer l, and $\epsilon^{(l)}$ is a learnable parameter.

The updated node representations from each GIN layer are sequentially applied by layer normalization, ReLU activation (except the last) and dropout. The lastly updated node representations are the final output from f_θ which are expressed as $H_{\mathcal{G}_\mathrm{M}}$ and $H_{\mathcal{G}_\mathrm{M}}$ for the original and decomposed molecular graph respectively.

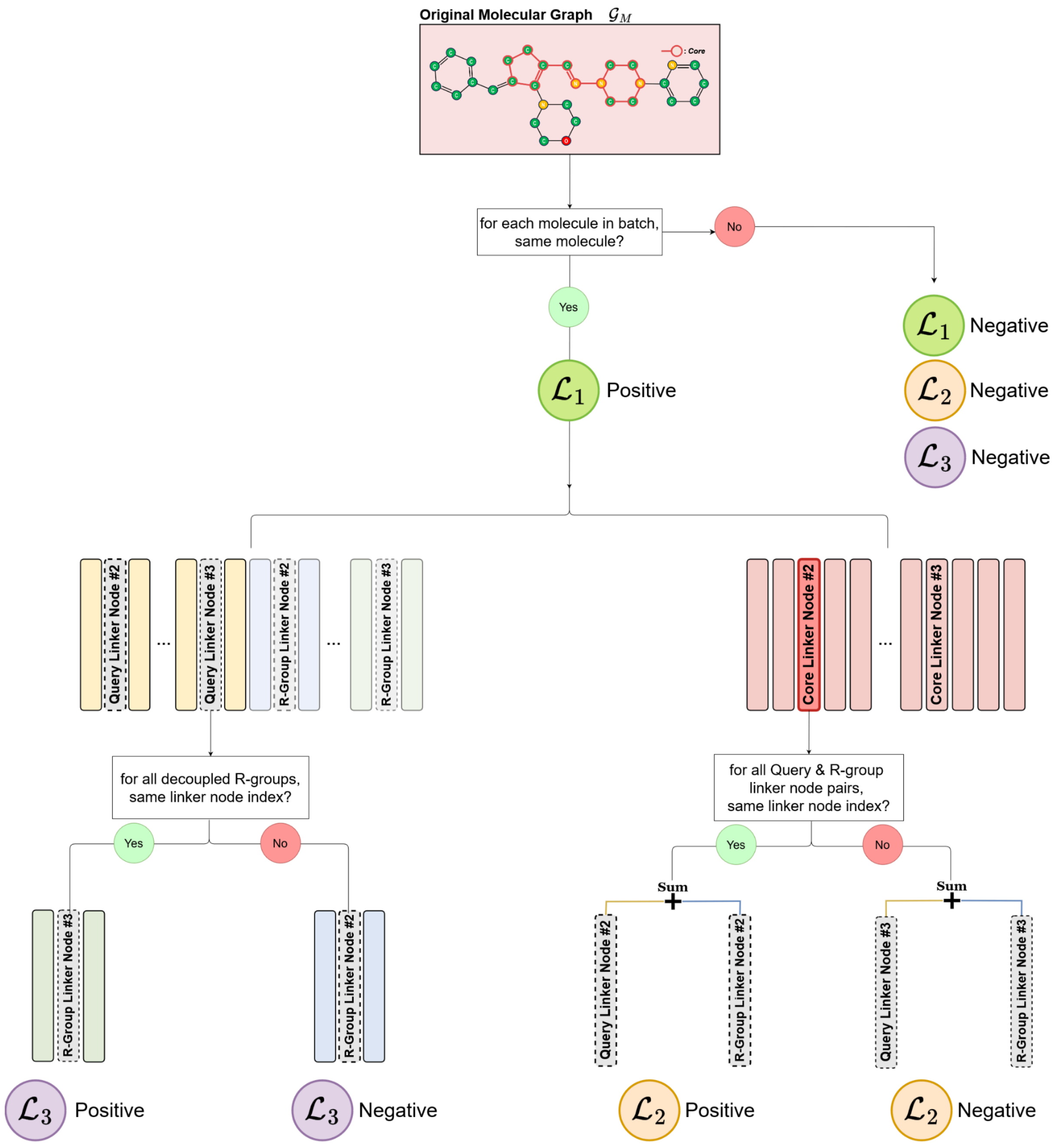

Illustration regarding the use of in-batch negatives while training MolPLA with three different loss objectives all based on InfoNCE loss. The number of negatives used in each loss term are all different since different molecules within the same batch may have different linker joints. Also, other R-Groups and Query & R-Group linker node pairs within same molecule are regarded as negatives.

S6: Illustration of the MolPLA's functionality in lead optimization

<table>
<tr><th colspan="2">Streptozocin (Core + Retrieved R-group)</th><th>Core</th><th>Retrieved R-group</th></tr>
<tr><td colspan="2">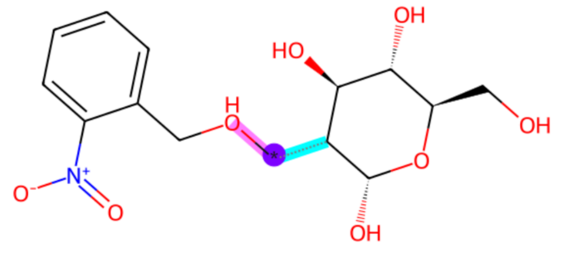</td><td>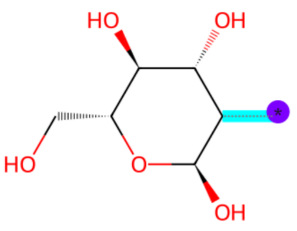</td><td>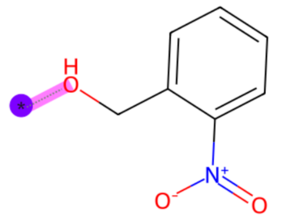</td></tr>
<tr><th colspan="4">Generated Molecules</th></tr>
<tr><td>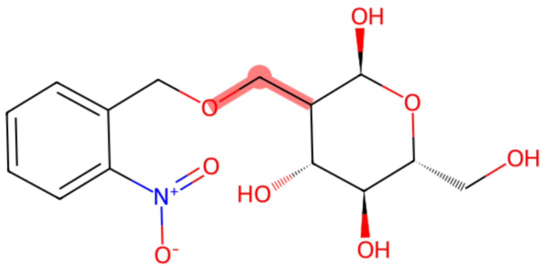
O=[N+]([O-])c1ccccc1COCC1[C@@H](O)[C@H](O)[C@@H](CO)O[C@@H]1O</td><td colspan="3">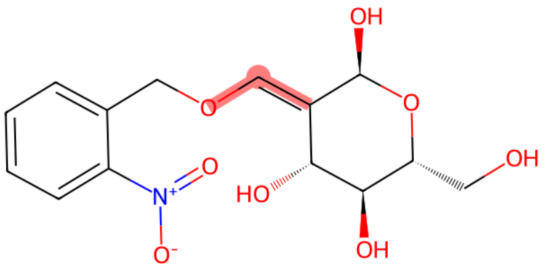
O=[N+]([O-])c1ccccc1COC=C1[C@@H](O)[C@H](O)[C@@H](CO)O[C@@H]1O</td></tr>
<tr><td>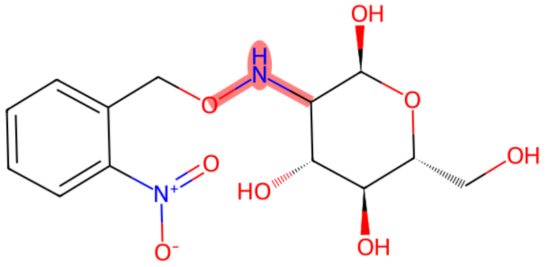
O=[N+]([O-])c1ccccc1CONC1[C@@H](O)[C@H](O)[C@@H](CO)O[C@@H]1O</td><td colspan="3">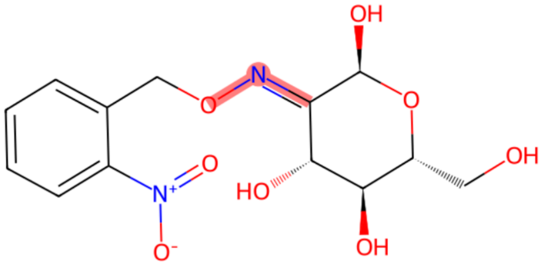
O=[N+]([O-])c1ccccc1CON=C1[C@@H](O)[C@H](O)[C@@H](CO)O[C@@H]1O</td></tr>
</table>

The linker node is first designated based on the node coloring results from the node representations of the query template (**core**) graph applied with PCA. The core graph containing the linker node with all its node-edge attributes masked is fed to MolPLA's pre-trained graph encoder. Among the encoded node representations, the linker node is exclusively selected, concatenated with the R-group condition vector and fed to MolPLA's query linker node projection head to **retrieve top nearest R-groups** in co-embedding space. For each R-group with its masked linker joint being re-attached to the query template, we invoke the enumeration process of all possible atom and bond features being used to "fill in" the masked attributes, and obtain re-attached molecules that are valid. These re-attached molecules are deemed as **generated molecules** and there may be multiple results since different combinations of unmasking atom and bond features can satisfy chemical rules.

S7: Visualization of Node Representations for Riluzole and Lasmiditan

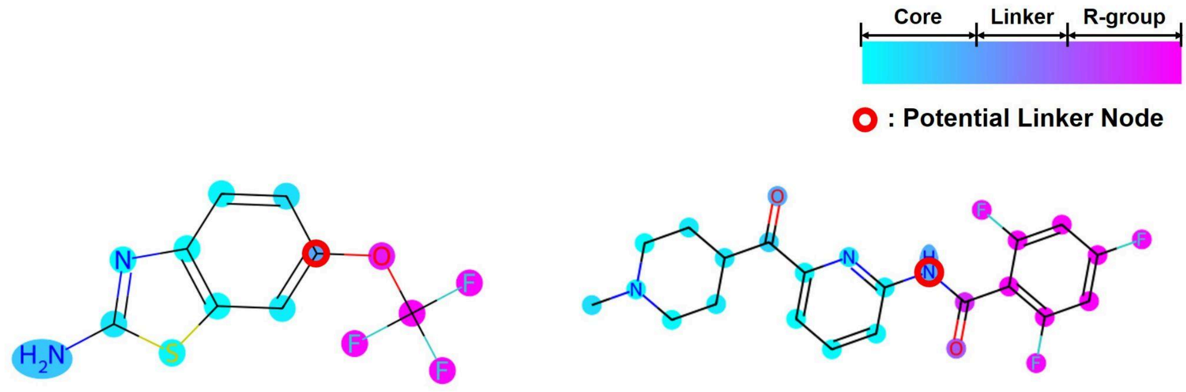

Riluzole **Lasmiditan**

S8: Distribution of QED and SAScores of molecule generated by MolPLA for Riluzole and Lasmiditan

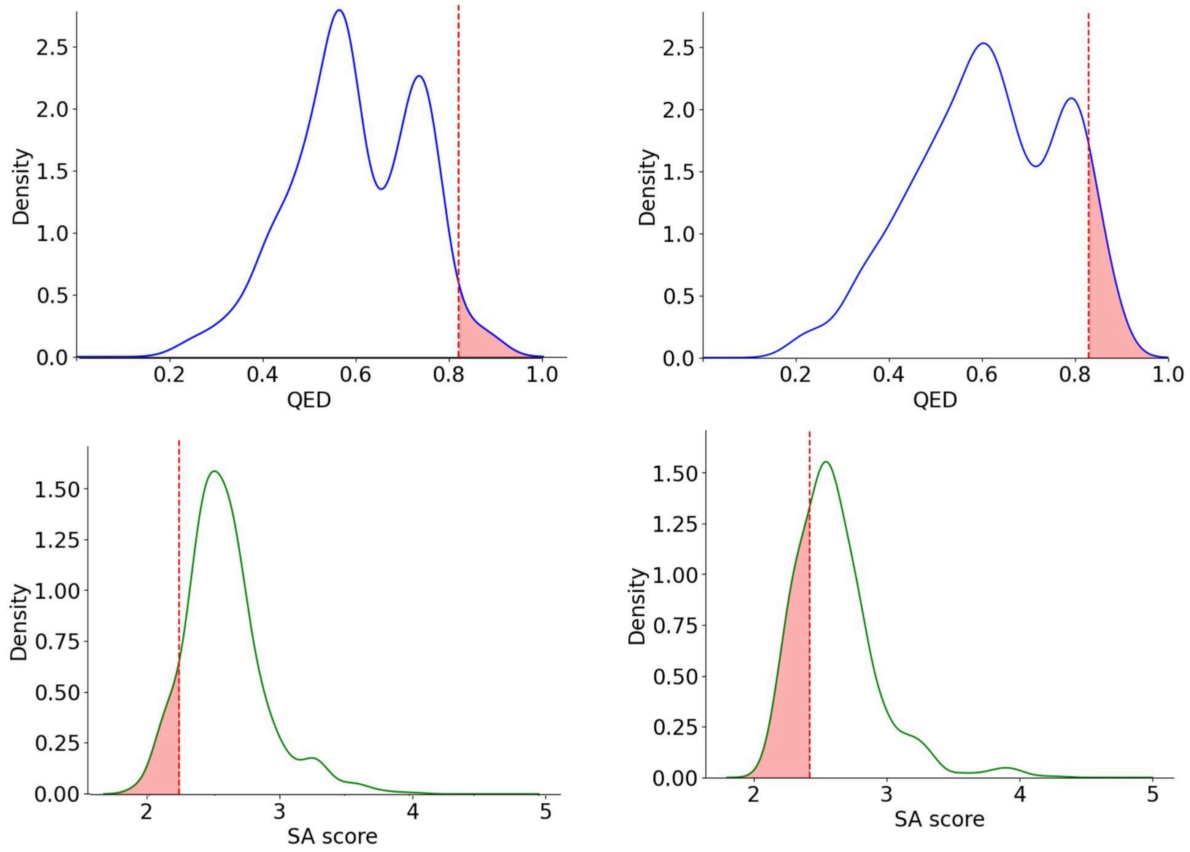

Riluzole **Lasmiditan**

S9: List of generated molecules for reference molecules Riluzole and Lasmiditan from MolPLA deployed in lead optimization scenario

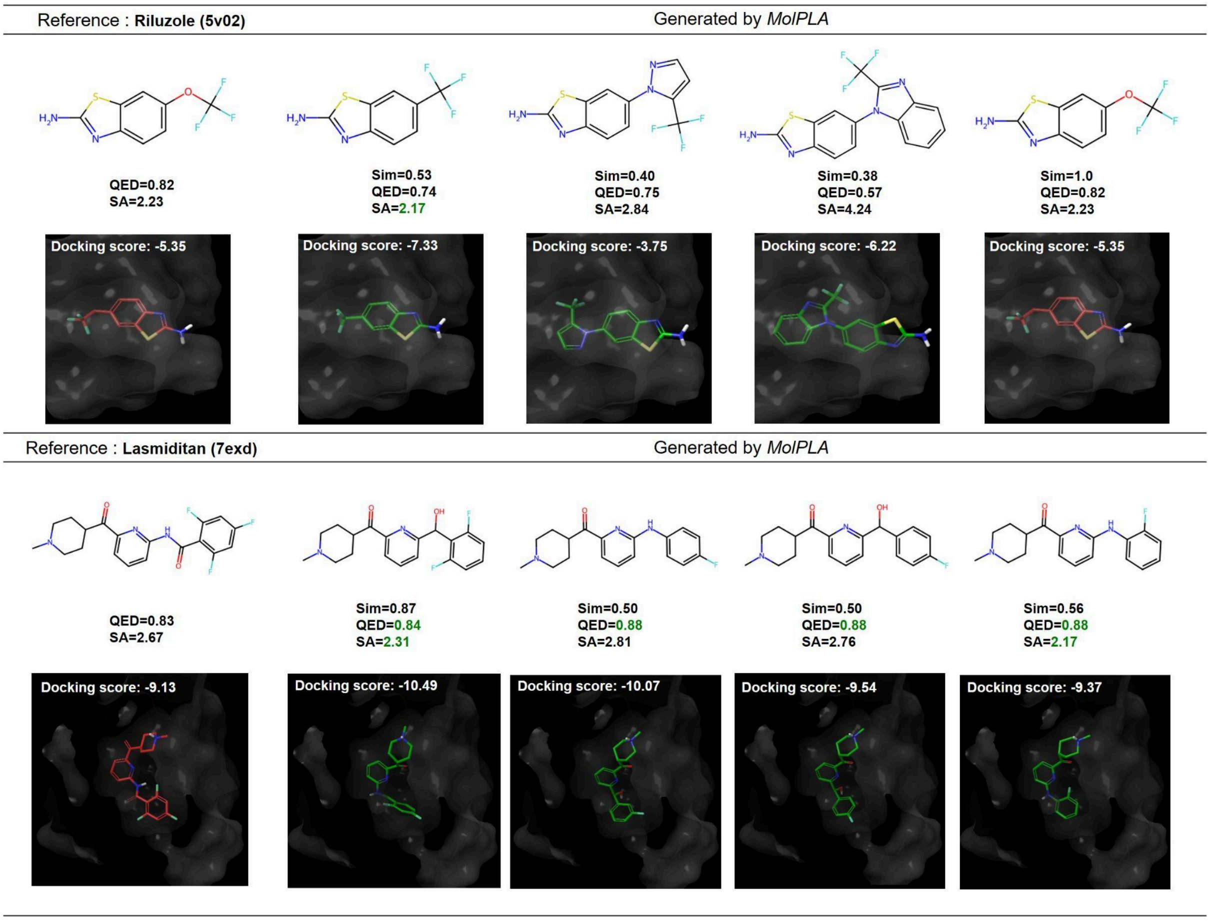

S10: Cross reference (Streptozocin, Capmatinib, Riluzole and Lasmiditan) analysis results for MolPLA's R-group Retrieval framework.

Query Template	R-group Substitution	Top 5 Retrieved R-groups (SMILES)
**Streptozocin**	**Streptozocin**	*~n1oc(=O)[nH]c1=O, *~n1ccc(=O)[nH]c1=O, *~n1ncc(=O)[nH]c1=O, *~n1c(=O)[nH]c(=O)n(C)c1=O, *~OCc1ccccc1[N+](=O)[O-]
	**Capmatinib**	*~OCc1ccccc1F, *~OCc1ccc(F)cc1, *~OCc1c(F)cccc1F, *~NOC(=O)c1ccccc1F, *~OCc1cc(F)ccc1-c1ccc(C)cc1
	**Riluzole**	*~OCc1c(F)cccc1F, *~NOC(=O)c1ccccc1F, *~OCc1cc(C(F)(F)F)cc(C(F)(F)F)c1, *~O[C@@H](C)c1cc(C(F)(F)F)cc(C(F)(F)F)c1, *~OC(C)c1cc(C(F)(F)F)cc(C(F)(F)F)c1
	**Lasmiditan**	*~OCc1ccccc1F, *~OCc1ccc(F)cc1, *~OCc1c(F)cccc1F, *~NOC(=O)c1ccccc1F, *~OCc1cc(F)ccc1-c1ccc(C)cc1
**Capmatinib**	**Streptozocin**	*~n1nnc(C(=O)/C=C/N(C)C)c1C, *~n1nc(C)c(C#N)c1N, *~n1nc(C)c(CN)c1C, *~n1nc(C)c(NC(=O)c2ccnn2C)c1C, *~n1nc(C)c(NC(=O)c2ccn(CC)n2)c1C
	**Capmatinib**	*~c1ccc(F)c(C(F)(F)F)c1, *~c1ccc(C(F)(F)F)c(F)c1, *~c1cccc(C(F)(F)F)c1, *~c1ccc(F)c2ccccc21, *~c1cc(C(F)(F)F)cc(C(F)(F)F)c1
	**Riluzole**	*~n1nc(C(F)F)cc1C(F)F, *~n1nc(C(F)(F)F)cc1C(F)(F)F, *~c1cc(C(F)(F)F)cc(C(F)(F)F)c1, *~n1c(C(F)(F)F)nc2cc(C(F)(F)F)ccc21, *~n1c(-c2ccc(F)cc2)nc2cc(C(F)(F)F)ccc21
	**Lasmiditan**	*~c1ccc(F)c(C(F)(F)F)c1, *~c1ccc(C(F)(F)F)c(F)c1, *~c1cccc(C(F)(F)F)c1, *~c1ccc(F)c2ccccc21, *~c1cc(C(F)(F)F)cc(C(F)(F)F)c1
**Riluzole**	**Streptozocin**	*~n1ccc(=O)cc1, *~n1nc(C)c[n+]1[O-], *~n1cccc1/C=N\O, *~n1cccc1/C=N/O, *~n1c(C)cc(/C=N/O)c1C
	**Capmatinib**	*~[NH2+]c1ccc(-c2ccccc2F)nn1, *~Nc1ncc(C(F)(F)F)c(NC2CCC2)n1, *~Nc1nc(-c2ccco2)cc(C(F)(F)F)n1, *~[NH2+]c1ncc(F)c(-c2cc(F)c3nc(C)n(C(C)C)c3c2)n1 *~Nc1ncc(F)c(-c2cc(F)c3nc(C)n(C(C)C)c3c2)n1
	**Riluzole**	*~c1c(F)c(F)c(C(F)(F)F)c(F)c1F, *~Oc1c(F)c(F)c(C(F)(F)F)c(F)c1F, *~n1c(C(F)(F)F)nc2cc(C(F)(F)F)ccc21, *~n1c(-c2ccc(F)cc2)nc2cc(C(F)(F)F)ccc21, *~[NH2+]c1ncc(F)c(-c2cc(F)c3nc(C)n(C(C)C)c3c2)n1
	**Lasmiditan**	*~[NH2+]c1ccc(-c2ccccc2F)nn1, *~Nc1ncc(C(F)(F)F)c(NC2CCC2)n1, *~Nc1nc(-c2ccco2)cc(C(F)(F)F)n1, *~[NH2+]c1ncc(F)c(-c2cc(F)c3nc(C)n(C(C)C)c3c2)n1 *~Nc1ncc(F)c(-c2cc(F)c3nc(C)n(C(C)C)c3c2)n1
**Lasmiditan**	**Streptozocin**	*~n1cc(C#N)c2c(N)ncnc21, *~n1c[n+](C)c2cc(C)c(C)cc21, *~n1c([NH3+])nc2ccccc21, *~n1cc[n+](CC=C)c1, *~n1cc([NH3+])cn1
	**Capmatinib**	*~[NH2+]c1ncc(-c2ccc(F)cc2)n1C, *~Nc1ncc(-c2ccc(F)cc2)n1C, *~Nc1nc2ccccc2n1Cc1ccc(F)cc1, *~[NH2+]c1ncc(F)c(-c2cc(F)c3nc(C)n(C(C)C)c3c2)n1, *~Nc1nc2c(c(=O)n(C)c(=O)n2C)n1Cc1ccc(F)cc1
	**Riluzole**	*~n1c(-c2ccc(F)cc2)noc1=O, *~n1cc(C(=O)O)c(=O)c2cc(F)c(F)c(F)c21, *~n1c(C(F)(F)F)nc2cc(C(F)(F)F)ccc21, *~n1c(-c2ccc(F)cc2)nc2cc(C(F)(F)F)ccc21, *~n1nnc(-c2cc(C(F)(F)F)cc(C(F)(F)F)c2)n1
	**Lasmiditan**	*~[NH2+]c1ncc(-c2ccc(F)cc2)n1C, *~Nc1ncc(-c2ccc(F)cc2)n1C, *~Nc1nc2ccccc2n1Cc1ccc(F)cc1, *~[NH2+]c1ncc(F)c(-c2cc(F)c3nc(C)n(C(C)C)c3c2)n1, *~Nc1nc2c(c(=O)n(C)c(=O)n2C)n1Cc1ccc(F)cc1

S11: Overview of MolPLA's Computational Efficiency: This includes the runtime data for generating 1,000 molecules using MolPLA's R-Group Retrieval framework, based on four reference molecules. Also detailed are the memory requirements and the hardware specifications used for these computations.

GPU Hardware: NVIDIA TITAN XP 12GB		
Reference Molecule	Run Time	GPU Memory
Capmatinib	11 minutes 34 seconds	< 2.5 GB
Streptozocin	14 minutes 9 seconds	< 2.5 GB
Riluzole	7 minutes 40 seconds	< 2.5 GB
Lasmiditan	3 minutes 20 seconds	< 2.5 GB
